# Stoppa Intrapelvic Approach Provides Good Functional Clinical Outcomes: Prospective Study with a Minimum Follow-up of One Year and Comparison with the Literature

**DOI:** 10.1055/s-0044-1785506

**Published:** 2024-06-22

**Authors:** Gabriel Canto Tomazini, Christiano Saliba Uliana, Marcelo Abagge, Henrique Reveilleau Fiorentin, Aramis Orlando Azevedo

**Affiliations:** 1 Departamento de Ortopedia e Traumatologia, Hospital do Trabalhador, Universidade Federal do Paraná, Curitiba, PR, Brasil

**Keywords:** acetabulum, fracture fixation, pelvis approach, Stoppa

## Abstract

**Objective** To describe the clinical and radiographic outcomes of a cohort of patients with acetabular fractures treated with the modified Stoppa approach.

**Methods** We conducted a prospective analysis of adult patients with acetabular fractures treated using the modified Stoppa approach from June 2020 to June 2021, with a minimum follow-up period of 12 months. The analysis included demographic, epidemiological, and perioperative data, as well as postoperative radiographic and functional outcomes.

**Results** The study included 15 cases, with 14 men (93.3%) and 1 woman (6.67%). A postoperative tomographic evaluation revealed an anatomical reduction in 50%, an imperfect reduction in 13.6%, and a poor reduction in 36.4% of the subjects. Regarding the functional scores, the Harris Hip Score ranged from 56 to 100, with a mean value of 92.5. The Majeed Pelvic Score classified the functional outcome as excellent in 36.5%, good in 40.6%, moderate in 18.7%, and poor in 4.2% of the cases.

**Conclusion** The present case series study demonstrated positive statistical relevance between reduction quality and functional outcomes and between the time until surgery and the reduction quality. The functional outcomes at a one-year of follow-up demonstrate that this approach can be an excellent alternative for anterior acetabulum fractures.

## Introduction

Since the study by Judet et al. 1 in the 1960s, with evidence later reinforced by Letournel and Judet 2 and Matta, 3 the fundamental principle for acetabular fracture treatment is its anatomical reduction with stable fixation, resulting in better short- and long-term functional outcomes. 1 3 4 Open reduction with internal fixation is the gold standard treatment. 5 6 Success largely depends on adequate exposure of the fracture focus 7 8 for better reduction and subsequent positioning of the synthesis materials.

Assessing alternative approaches, Cole and Bolhofner 9 and Hirvensalo et al. 10 developed 5 7 11 12 an intrapelvic route to assure a wide approach to the true pelvis. 5 7 8 9 13 After its development, the authors found similarities in the method proposed by Stoppa et al. 14 in 1984 to manage complicated inguinal hernias. 9 12 This method enables direct visualization of the acetabulum, including the quadrilateral lamina, 4 5 6 8 the body of the pubis, the superior ramus, the pubic root, and the medial aspect of the posterior and anterior columns of the sacroiliac joint. 5 7 8 This exposure also enables plate configurations not feasible with traditional approaches 7 9 (such as the infrapectineal plate 11 ). However, this method may entail complications, such as obturator nerve damage, corona mortis, rectus abdominis atrophy, and peritoneal injury. 4 7

Today, the modified Stoppa approach may be an alternative to manage anterior acetabular fractures. Even with broad visualization of the true pelvis, 60% to 83% 6 of the cases may require a combination with other approaches for adequate reduction and fixation of some types of fracture, 6 7 12 including the lateral window ilioinguinal 6 7 and the Kocher-Langenbeck approaches. 6 8

The present study aimed to describe the clinical and radiographic outcomes of a cohort of patients with acetabular fractures treated with the modified Stoppa approach.

## Materials and Methods

The institutional Ethics in Research Committee analyzed and approved the design of the present study under number CAAE 52764821.4.0000.5225. The prospective analysis included data from patients with acetabular fractures who underwent open reduction and internal fixation (ORIF) using the modified Stoppa approach in a university hospital from June 2020 to June 2021. The inclusion criteria were subjects over 18 years old with a minimum postoperative follow-up period of 12 months. The exclusion criteria were cases of osteoporotic/pathological fractures.

We selected the modified Stoppa approach for cases with acetabular anterior column involvement, either isolated or in combined fracture patterns. Thus, according to the Letournel and Judet classification, 2 fractures of the following types were included: anterior column, transverse, anterior column with posterior hemitransverse, T-shaped, and double-column fracture. For transverse fractures, we chose the anterior approach when the anterior column presented a higher deviation than the posterior column.

The data collected during the study included gender; age; trauma mechanism; preoperative Letournel 15 classification; postoperative reduction quality per the Matta criteria 3 (evaluated by two orthopedic doctors specialized in hips), through radiographs and computed tomography (CT) scans; days from admission to definitive approach; surgical time; blood loss; need for transfusion; other concomitant approaches; associated injuries; and peri- and postoperative complications. The categories of the Matta 3 classification were the following: 1 (anatomical reduction), 2 (imperfect reduction, ranging from 1 mm to 3 mm), and 3 (poor reduction, greater than 3 mm). The patients answered functional questionnaires at one year of outpatient follow-up: the modified Harris Hip Score (HHS) and the Majeed Pelvic Score (MPS).

**Fig. 1:**
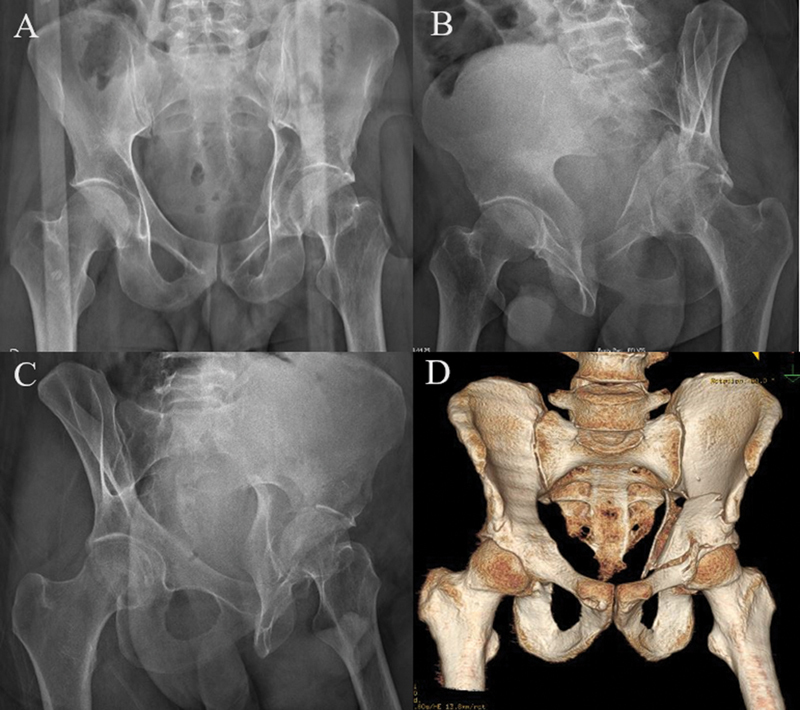
( **A** ) Anteroposterior (AP) radiograph. ( **B** ) Obturator oblique radiograph. ( **C** ) Alar radiograph. ( **D** ) Three-dimensional (3D) computed tomography showing double column fracture of the left acetabulum.

**Fig. 2:**
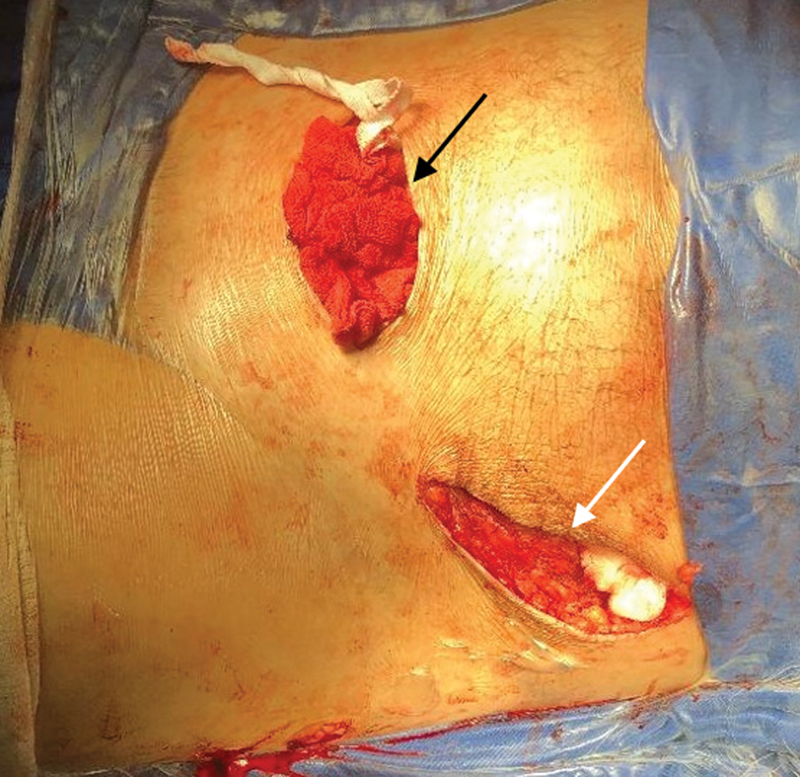
Appearance of the approaches used in the study. **Black arrow:** Modified Stoppa approach. **White arrow:** Ilioinguinal lateral window.

The routine preoperative and postoperative examinations included pelvic radiographs in anteroposterior and Judet views, axial CT of the pelvis with three-dimensional reconstruction, and laboratory tests ( Fig. 1 ) In the surgical center, we always perform trichotomy and image checking with fluoroscopy on a radiolucent table before starting the procedure. We prepared the entire lower limb on the affected side to have free mobility for flexion and traction to assist in reduction maneuvers during surgery. The modified Stoppa approach was always the first to be performed. Other approaches were performed sequentially during the procedure according to need and preoperative planning ( Fig. 2 ).

Motor physical therapy started early with free range of motion. Next, the authors instructed the patients to only bear the proprioceptive load on the affected limb aided by a walker. Sequential outpatient return for weight progression occurred at 2 weeks, 8 weeks, 4 months, 6 months, and 1 year postoperatively.

The collected data were tabulated and statistically analyzed using the Pearson correlation coefficient for normally distributed continuous variables (confirmed by the Shapiro-Wilk test; *p* -value > 0.05) and Kendall tau correlations for other variables for inferences.

## Results

**Fig. 3:**
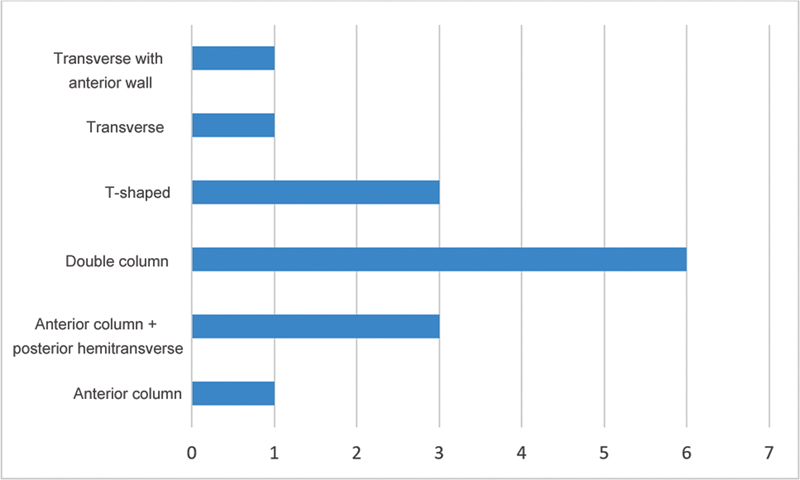
Frequency of fractures according to the Letournel classification.

After applying the inclusion and exclusion criteria, 15 cases were analyzed, including 14 men (93.3%) and 1 woman (6.67%). Table 1 shows the age, operative time, and days until surgery. Fig. 3 shows the frequency of fractures according to the Letorunel and Judet 2 classification.

**Table 1:** Case profile

	Mean	Standard deviation	Minimum value	Maximum value
Age (years)	35.4	±11.879	20	55
Surgical time (minutes)	221	±77.78	90	390
Days until surgery	11.067	±8.093	4	33

The postoperative tomographic evaluation revealed an anatomical reduction in 50%, an imperfect reduction in 13.6%, and a poor reduction in 36.4% of the subjects. Regarding the functional scores, the HHS ranged from 56 to 100, with a mean value of 92.5. The MPS classified the functional outcome as excellent in 36.5%, good in 40.6%, moderate in 18.7%, and poor in 4.2% of the cases.

The correlation regarding time from admission to definitive surgery and reduction quality was statistically significant. The MPS and HHS positively correlated with reduction quality ( Table 2 ).

**Table 2:** Kendall correlations

	Kendall τ-B	*p*
Time until surgery *versus* surgical time	0.311	0.126
Surgical time *versus* reduction quality	0.241	0.283
HHS *versus* MPS (1 year)	0.404	0.036
**Reduction quality** ***versus*** **HHS**	− **0.602**	**0.006**
**Reduction quality** ***versus*** **MPS**	− **0.577**	**0.006**
**Reduction quality** ***versus*** **time until surgery**	**0.544**	**0.007**

**Abbreviations:** HHS, Harris Hip Score; MPS, Majeed Pelvic Score.

Of the 15 cases, some presented postoperative complications: 4 had obturator nerve praxis, and 1 had lateral cutaneous nerve praxis, which resolved during follow-up. In addition, there were four surgical site infections (one using the modified Stoppa approach, later progressing to septic arthritis), one corona mortis injury (requiring a joint approach with the service's vascular surgery team at the same operative time), and a case of late complication with incisional hernia. Soni et al. 16 reported obturator nerve injury as the most frequent complication, with complete resolution in 95% of THE cases within 3 to 6 months. Among late complications, osteoarthritis was the most common. In the present series, 27.7% of the patients presented obturator nerve praxis, all resolving completely during the 1-year follow-up.

**Fig. 4:**
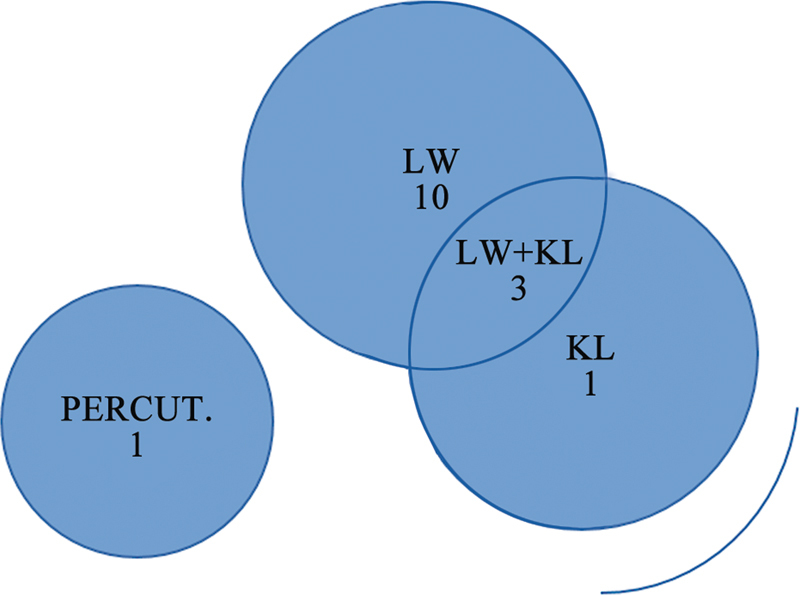
Associated approaches.

In total, 13 out of the 15 cases required a lateral window, and 4, a Kocher-Langenbeck approach (including 3 associated with a lateral window). One case used a percutaneous approach ( Fig. 4 ). Most studies reported treatment with a single Stoppa approach in most cases. Guo et al. 8 used a single Stoppa approach in 59% of the patients and the Stoppa plus the posterior Kocher Langenbeck approach in 40% of the patients.

The estimated procedural average bleeding was of 1,411 mL (range: 400 mL to 3,525 mL), consistent with the average value of 1,376 mL reported by Laflamme et al. 17 Six cases required an intraoperative blood transfusion, and one patient received a postoperative transfusion. There were no severe hemorrhage-related complications in the present case series.

Finally, eight cases presented associated injuries, including three non-orthopedic injuries (aortic injury, epidural hematoma, and tension hemothorax). Orthopedic injuries to the upper limbs were more frequent and observed in five cases.

Surgery time and intraoperative bleeding presented a positive correlation (Pearson r = 0.712; *p* = 0.003; 95% confidence interval [95%CI] = 0.314 to 0.897). There was no correlation between surgery and reduction quality (Kendall τ; *p* = 0.283) ( Table 2 ) or time until surgery (Kendall τ; *p* = 0.126). However, longer times until surgery had a statistical significance with lower reduction quality ( *p* = 0.007). The MPS and HHS correlated with the reduction quality (Kendall τ; *p* < 0.006 for both scores).

## Discussion

**Fig. 5:**
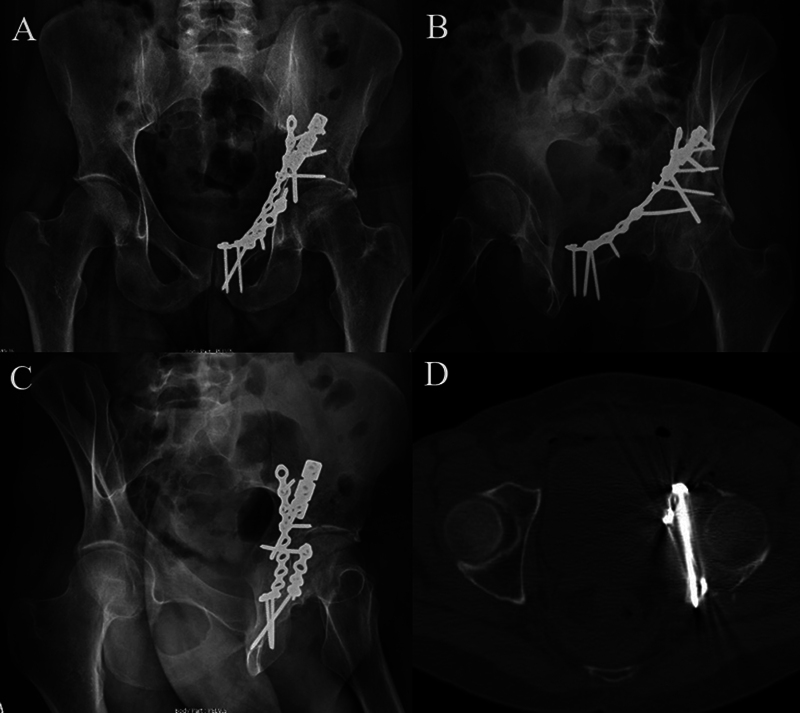
( **A** ) Postoperative AP radiograph. ( **B** ) Postoperative oblique obturator radiograph. ( **C** ) Postoperative alar radiograph. ( **D** ) Axial section of the postoperative computed tomography scan demonstrating the angle of attack and positioning of the interfragmentary screw fixing the anterior column to the posterior column.

**Fig. 6:**
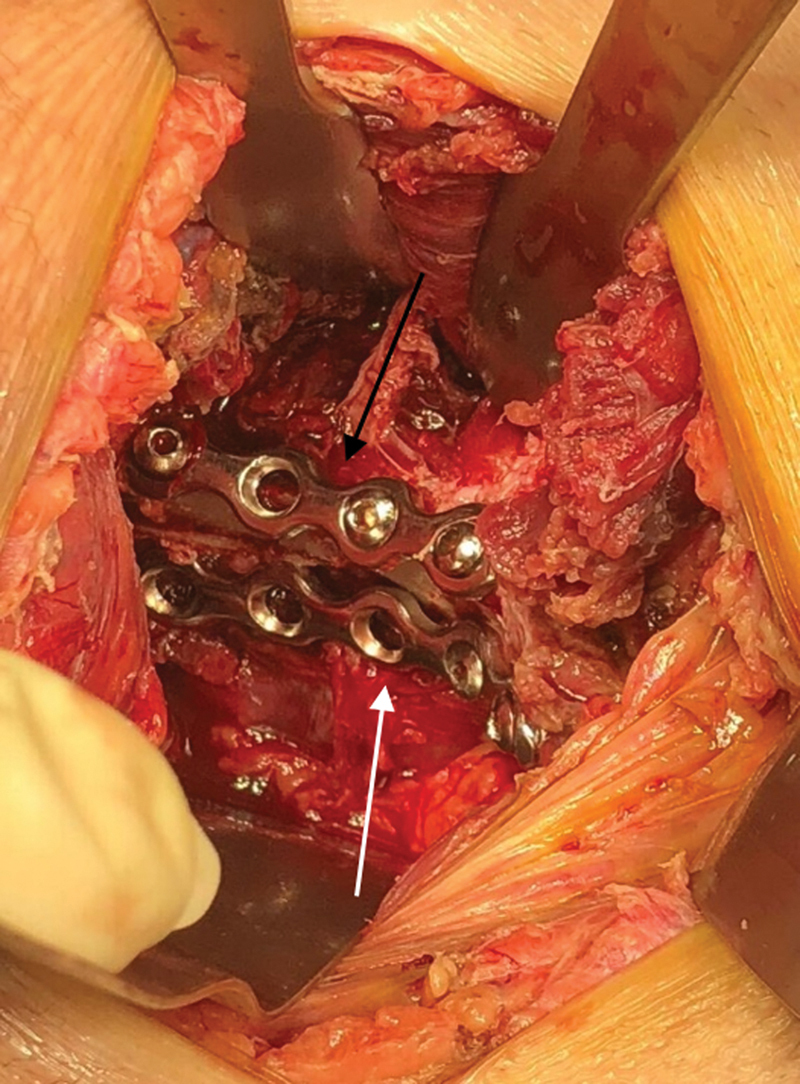
Intraoperative appearance. **Black arrow:** Suprapectineal plate. **White arrow:** Infrapectineal plate.

The modified Stoppa approach enabled a good attack angle for maneuvers to reduce the quadrilateral blade and fixation ( Fig. 5 ) either with interfragmentary screws or anti-shear plates. ( Fig. 6 ). Lateral femoral traction also helps fracture reduction with this approach, and a Schanz pin in the femoral neck decreases pressure on the blade and enables its reduction.

The present study had a relatively low number of cases using exclusively the Stoppa approach. The lateral window of the associated ilioinguinal approach seems a good concomitant option. The increasing learning curve will probably result in lower use of the posterior Kocher-Langenbeck approach at the same time, since studies with larger samples present a lower percentage of concomitant use of this route with the modified Stoppa approach.

We believe that maintaining the integrity of the inguinal canal results in lower rates of dissection of soft tissues and, consequently, a lower chance of infection. The time between trauma and the definitive surgical approach is a significant factor because fractures older than 15 days tend to present lower levels of mobilization and, as a result, difficult reduction. Sometimes, these injuries require more than two approaches, with increased surgical time and worse functional outcomes.

## Conclusion

We concluded that the modified Stoppa approach provided good functional and clinical outcomes, demonstrated by an average HHS of 92.5 and 77.1% of excellent or good results on the MPS. The case series study demonstrated positive statistical relevance between the reduction quality and functional outcomes and between the time until surgery and the reduction quality. At the one-year follow-up, this approach can be an excellent alternative for anterior acetabular fractures.

## References

[1] Judet R Judet J Letournel E Fractures of the acetabulum: classification and surgical approaches for open reduction. preliminary report J Bone Joint Surg Am 1964 46 1615 1646 14239854

[2] Letournel E Judet R Fractures of the Acetabulum 2nd ed. New York, NY Springer-Verlag 1993

[3] Matta J M Fractures of the acetabulum: accuracy of reduction and clinical results in patients managed operatively within three weeks after the injury J Bone Joint Surg Am 1996 78 11 1632 1645 8934477

[4] Kilinc C Y Acan A E Gultac E Kilinc R M Hapa O Aydogan N H Treatment results for acetabulum fractures using the modified Stoppa approach Acta Orthop Traumatol Turc 2019 53 01 6 14 30558866 10.1016/j.aott.2018.11.003 PMC6424668

[5] Liu Y Yang H Li X Yang S H Lin J H Newly modified Stoppa approach for acetabular fractures Int Orthop 2013 37 07 1347 1353 23681609 10.1007/s00264-013-1920-7 PMC3685676

[6] Yao S Chen K Ji Y Supra-ilioinguinal versus modified Stoppa approach in the treatment of acetabular fractures: reduction quality and early clinical results of a retrospective study J Orthop Surg Res 2019 14 01 364 31727107 10.1186/s13018-019-1428-y PMC6854625

[7] Meena S Sharma P K Mittal S Sharma J Chowdhury B Modified Stoppa Approach versus Ilioinguinal Approach for Anterior Acetabular Fractures; A Systematic Review and Meta-Analysis Bull Emerg Trauma 2017 5 01 6 12 28246617 PMC5316130

[8] Guo H Z He Y F He W Q Modified stoppa approach for pelvic and acetabular fracture treatment Acta Ortop Bras 2019 27 04 216 219 31452623 10.1590/1413-785220192704188933 PMC6699384

[9] Cole J D Bolhofner B R Acetabular fracture fixation via a modified Stoppa limited intrapelvic approach. Description of operative technique and preliminary treatment results Clin Orthop Relat Res 1994 305 112 123 8050220

[10] Hirvensalo E Lindahl J Böstman O A new approach to the internal fixation of unstable pelvic fractures Clin Orthop Relat Res 1993 297 28 32 8242945

[11] Balbachevsky D Pires R Faloppa F Reis F Tratamento das fraturas da pelve e acetábulo pela via de Stoppa modificada Acta Ortop Bras 2006 14 04 190 192

[12] Isaacson M J Taylor B C French B G Poka A Treatment of acetabulum fractures through the modified Stoppa approach: strategies and outcomes Clin Orthop Relat Res 2014 472 11 3345 3352 24420164 10.1007/s11999-014-3460-x PMC4182379

[13] Wang X J Zhang Z H Ilioinguinal approach versus Stoppa approach for open reduction and internal fixation in the treatment of displaced acetabular fractures: A systematic review and meta-analysis Chin J Traumatol 2017 20 04 229 234 28709737 10.1016/j.cjtee.2017.01.005 PMC5555276

[14] Stoppa R E Rives J L Warlaumont C R Palot J P Verhaeghe P J Delattre J F The use of Dacron in the repair of hernias of the groin Surg Clin North Am 1984 64 02 269 285 6233733 10.1016/s0039-6109(16)43284-6

[15] Letournel E Acetabulum fractures: classification and management Clin Orthop Relat Res 1980 151 81 106 7418327

[16] Soni A Gupta R Sen R Modified Stoppa Approach for Acetabulum Fracture: A Review Rev Bras Ortop 2019 54 02 109 117 10.1016/j.rboe.2017.09.006 PMC6510579 31363255

[17] Laflamme G Y Hebert-Davies J Rouleau D Benoit B Leduc S Internal fixation of osteopenic acetabular fractures involving the quadrilateral plate Injury 2011 42 10 1130 1134 21156315 10.1016/j.injury.2010.11.060

