# Supplementary material for: Stoppa Intrapelvic Approach Provides Good Functional Clinical Outcomes: Prospective Study with a Minimum Follow-up of One Year and Comparison with the Literature
Source: Rev Bras Ortop (Sao Paulo). 2024 Jun 22;59(3):e443–8. doi: 10.1055/s-0044-1785506 (PMC11193587; doi:10.1055/s-0044-1785506)
Supplement: Related texts

# Research article: Acesso intrapélvico de Stoppa proporciona bons resultados clínicos funcionais: Estudo prospectivo com seguimento mínimo de um ano e comparação com a literatura

**Keywords:** abordagem pélvica, acetábulo, fixação de fratura, Stoppa

## Abstract

**Objetivo** Descrever os resultados clínicos e radiográficos de uma coorte de pacientes com fraturas de acetábulo tratados com o acesso de Stoppa modificado.

**Métodos** Foi realizada uma análise prospectiva de pacientes adultos com fraturas de acetábulo tratados pela via de Stoppa modificada de junho de 2020 a junho de 2021 e com seguimento mínimo de 12 meses. Foram analisados dados demográficos, epidemiológicos e perioperatórios, e resultados radiográficos e funcionais pós-operatórios.

**Resultados** Foram estudados 15 casos, sendo 14 homens (93,3%) e 1 mulher (6,67%). Na avaliação tomográfica pós-operatória, redução anatômica foi observada em 50% dos casos, imperfeita, em 13,6%, e ruim, em 36,4%.

Nos escores funcionais, encontramos uma variação de 56 a 100, com média de 92,5 no Harris Hip Score. No Majeed Pelvic Score, o resultado funcional foi excelente em 36,5% dos casos, bom, em 40,6%, moderado, em 18,7%, e ruim, em 4,2%.

**Conclusão** O estudo da série de casos demonstrou relevância estatística positiva entre a qualidade da redução e os desfechos funcionais, assim como entre o tempo até a cirurgia e a qualidade da redução. Os resultados funcionais no seguimento de um ano demonstram que o uso dessa via pode ser uma excelente alternativa para as fraturas anteriores do acetábulo.

## Introdução

Desde os trabalhos de Judet et al. 1 na década de 1960, com evidências reforçadas posteriormente por Letournel e Judet 2 e Matta, 3 considera-se princípio fundamental do tratamento das fraturas de acetábulo sua redução anatômica com fixação estável, que apresenta resultados funcionais melhores no curto e longo prazos, 1 3 4 sendo o padrão-ouro a redução aberta com fixação interna. 5 6 O sucesso depende, em boa parte, da adequada exposição do foco da fratura 7 8 para melhor redução e subsequente posicionamento dos materiais de síntese.

No estudo de alternativas de abordagens, Cole e Bolhofner 9 e Hirvensalo et al *.* 10 desenvolveram 5 7 11 12 uma via intrapélvica que garante amplo acesso à pelve verdadeira. 5 7 8 9 13 Após isso, os autores encontraram similaridades na via proposta por Stoppa et al. 14 em 1984 para o manejo de hérnias inguinais complicadas. 9 12 Assim, se permite a visualização direta do acetábulo, incluindo a lâmina quadrilátera, 4 5 6 8 o corpo do púbis, o ramo superior, a raiz púbica, e do aspecto medial das colunas posterior e anterior da articulação sacroilíaca. 5 7 8 Essa exposição também permite configurações de placa que não são possíveis nas abordagens tradicionais 7 9 (como placa infrapectínea 11 ). Por outro lado, também apresenta riscos de complicações, como lesão do nervo obturador, da *corona* *mortis* , atrofia de reto abdominal e lesão peritoneal. 4 7

Atualmente, a via de Stoppa modificada pode ser uma alternativa para o manejo de fraturas anteriores do acetábulo. Mesmo com ampla visualização da pelve verdadeira, em 60% a 83% 6 dos casos, pode ser necessária associação de outros acessos para a redução e a fixação adequadas de alguns tipos de fratura, 6 7 12 como a janela lateral do acesso ilioinguinal 6 7 ou a via de Kocher-Langenbeck. 6 8

O objetivo deste estudo é descrever os resultados clínicos e radiográficos de uma coorte de pacientes com fraturas de acetábulo tratados pelo acesso de Stoppa modificado.

## Materiais e Métodos

O desenho deste estudo foi analisado e aprovado pelo Comitê de Ética em Pesquisa da instituição (CAAE: 52764821.4.0000.5225). Foi realizada uma análise prospectiva de dados de pacientes com fraturas de acetábulo submetidos à redução aberta e fixação interna (RAFI) pelo acesso de Stoppa modificado em hospital universitário de junho de 2020 a junho de 2021. Foram incluídos no estudo os pacientes maiores de 18 anos com acompanhamento mínimo de 12 meses após o tratamento cirúrgico. Foram excluídos casos de fraturas osteoporóticas/patológicas.

A abordagem de Stoppa modificada foi escolhida nos casos em que havia comprometimento da coluna anterior acetabular, tanto na forma isolada quanto nos padrões de fraturas combinadas. Assim, segundo a classificação de Letournel e Judet, 2 foram incluídas fraturas dos tipos: coluna anterior, transversa, coluna anterior com hemitransversa posterior, em T e dupla coluna. Nas fraturas transversas, o acesso anterior foi escolhido quando a coluna anterior apresentava maior desvio em relação ao desvio da coluna posterior.

Os dados coletados durante o estudo foram: gênero; idade; mecanismo do trauma; classificação pré-operatória conforme Letournel; 15 qualidade da redução pós-operatória segundo os critérios de Matta 3 (avaliada por dois médicos ortopedistas especialistas em quadril), por meio de radiografias e tomografias; dias entre internação e abordagem definitiva; tempo cirúrgico; perda sanguínea; necessidade de transfusão; outros acessos concomitantes; lesões associadas; e complicações peri e pós-operatórias. Para as análises, a classificação de Matta 3 foi considerada categoricamente como: 1 (anatômica), 2 (imperfeita: 1 mm a 3mm) e 3 (ruim: maior do que 3 mm). Foram aplicados dois escores funcionais após um ano de acompanhamento ambulatorial: o Harris Hip Score (HHS) modificado e Majeed Pelvic Score (MPS).

A rotina de exames pré e pós-operatórios incluiu radiografias de pelve em incidência anteroposterior e nas incidências de Judet, e tomografia axial computadorizada de pelve com reconstrução tridimensional, além de exames laboratoriais ( Fig. 1 ). No centro cirúrgico, são sempre realizadas a tricotomia e a conferência das imagens com fluoroscopia em mesa radiotransparente, antes do início do procedimento, sendo preparado todo o membro inferior do lado acometido para ter mobilidade livre para flexão e tração, para auxiliar nas manobras de redução durante a cirurgia. O acesso de Stoppa modificado sempre foi o primeiro a ser realizado. Outros acessos eram realizados na sequência durante o procedimento conforme a necessidade e o planejamento pré-operatório ( Fig. 2 ).

**Fig. 1:** ( **A** ) **:** Radiografia em incidência anteroposterior (AP). ( **B** ) Radiografia em obturatriz. **C** ) Radiografia em alar. ( **D** ) Tomografia tridimensional (3D) que evidencia fratura em dupla coluna do acetábulo esquerdo.

**Fig. 2:** Aspecto dos acessos utilizados. **Seta preta** : acesso de Stoppa modificado. **Seta branca** : janela lateral do ilioinguinal.

A fisioterapia motora é iniciada precocemente com amplitude de movimento livre; o paciente então é orientado a realizar apenas carga proprioceptiva no membro acometido com o auxílio de andador. O retorno ambulatorial sequencial para a progressão da carga se deu com 2 semanas, 8 semanas, 4 meses, 6 meses e 1 ano de pós-operatório.

Os dados coletados foram tabulados e analisados estatisticamente utilizando coeficiente de correlação de Pearson para variáveis contínuas de distribuição normal (confirmadas pelo teste de Shapiro-Wilk; *p* > 0,05) e correlações de Tau de Kendall para as demais variáveis para inferências.

## Resultados

No período analisado, após aplicados os critérios de inclusão e exclusão, foram estudados 15 casos, sendo 14 homens (93,3%) e 1 mulher (6,67%). Outros dados, como idade, tempo operatório e dias até a cirurgia estão contidos na Tabela 1 , e a frequência de fraturas conforme classificação de Letorunel e Judet, 2 na Fig. 3 .

**Tabela 1:** Perfil dos casos

	Média	Desvio padrão	Mínimo	Máximo
Idade (anos)	35,4	±11,879	20	55
Tempo operatório (minutos)	221	±77,78	90	390
Dias até cirurgia	11,067	±8,093	4	33

**Fig. 3:** Frequência das fraturas conforme Letournel.

Na avaliação tomográfica pós-operatória, redução anatômica foi observada em 50% dos casos, imperfeita, em 13,6% e ruim, em 36,4%. Nos scores funcionais, encontramos uma variação de 56 a 100, com média de 92,5 no HHS. No MPS, o resultado funcional foi excelente em 36,5% dos casos, bom em 40,6%, moderado em 18,7% e ruim em 4,2%.

A correlação do tempo entre internamento e cirurgia definitiva e a qualidade da redução foi estatisticamente significativa. O MPS e o HHS se correlacionaram positivamente com a qualidade da redução ( Tabela 2 ).

**Tabela 2:** Correlações de Kendall

	τ b de Kendall	*p*
Tempo até procedimento *versus* tempo operatório	0,311	0,126
Tempo operatório *versus* qualidade da redução	0,241	0,283
HHS *versus* MPS (1 ano)	0,404	0,036
**Qualidade da redução** ***versus*** **HHS**	− **0,602**	**0,006**
**Qualidade da redução** ***versus*** **MPS**	− **0,577**	**0,006**
**Qualidade da redução** ***versus*** **tempo até o procedimento**	**0,544**	**0,007**

**Abreviaturas:** HHS, Harris Hip Score; MPS, Majeed Pelvic Score.

Entre os 15 casos, houve algumas complicações pós-operatórias: 4 praxias do nervo Obturador e 1 de nervo cutâneo lateral, que obtiveram resolução no seguimento; 4 infecções de sítio cirúrgico (das quais apenas 1 no acesso de Stoppa modificado, que complicou tardiamente com artrite séptica); 1 lesão de *corona* *mortis* , que necessitou de abordagem conjunta com a equipe de cirurgia vascular do serviço, no mesmo tempo operatório; e 1 caso de complicação tardia com hérnia incisional. A lesão do nervo obturador foi a complicação mais frequente apontada por Soni et al., 16 mas com resolução completa em 95% dos casos em 3 a 6 meses. Em relação às complicações tardias, a artrose foi a mais comum. Em nossa série, 27,7% dos pacientes apresentaram praxia do nervo obturador, todos com resolução completa no acompanhamento de 1 ano.

Ao todo, 13 dos 15 casos necessitaram do uso da janela lateral e 4, da via de Kocher-Langenbeck (sendo 3 associados à janela lateral). Em um dos casos, foi utilizada abordagem percutânea ( Fig. 4 ). A maioria dos estudos demonstraram o tratamento com acesso único de Stoppa em grande parte dos casos: no estudo de Guo et al., 8 em 59% dos pacientes, seguido de 40% de Stoppa mais acesso posterior de Kocher Langenbeck;

**Fig. 4:** Acessos associados.

A média estimada de sangramento nos procedimentos foi de 1.411 mL (variação: 400 mL a 3.525 mL), valor próximo ao encontrado por Laflamme et al., 17 com 1.376 mL de média. Foi necessária hemotransfusão intraoperatória em seis casos e, somente em um, pós-operatória. Não houve complicações graves relacionadas à hemorragia na série de casos.

Por fim, houve oito casos de lesões associadas. Destas, três não foram ortopédicas: lesão de aorta, hematoma epidural e hemotórax hipertensivo. Lesões ortopédicas de membros superiores foram mais frequentes, e estiveram presentes em cinco casos.

Foi encontrada correlação positiva (r de Pearson = 0,712; *p* = 0,003; intervalo de confiança de 95% [IC95%] 0,314 a 0,897) para tempo de cirurgia com sangramento intraoperatório. O tempo de cirurgia não teve correlação com a qualidade da redução (τ de Kendall; *p* = 0,283 Tabela 2 ) ou com o tempo até a cirurgia (τ de Kendall; *p* = 0,126). Porém, maiores tempos até a abordagem cirúrgica obtiveram significância estatística para piores qualidades de redução ( *p* = 0,007). O MPS e o HHS correlacionaram com a qualidade da redução (τ de Kendall; *p* < 0,006 para ambos).

## Discussão

Percebemos que o acesso de Stoppa modificado permite um bom ângulo de ataque tanto para manobras de redução da lâmina quadrilátera quanto para a fixação dela ( Fig. 5 ), seja com parafusos interfragmentários ou placas anticisalhantes ( Fig. 6 ). Outra manobra que ajuda bastante na redução das fraturas tratadas com esse acesso é a tração lateral do fêmur, com o auxílio de um pino de Schanz no colo femoral, para diminuir a pressão sobre a lâmina e permitir a sua redução.

**Fig. 5:** ( **A** ) Radiografia pós-operatória em AP. ( **B** ) Radiografia pós-operatória em obturatriz. ( **C** ) Radiografia pós-operatória em alar. ( **D** ) Corte axial da tomografia pós-operatória que demonstra ângulo de ataque e posição de parafuso interfragmentário fixando a coluna anterior à coluna posterior.

**Fig. 6:** Aspecto intraoperatório **. Seta preta:** placa suprapectínea. **Seta branca** : placa infrapectínea.

No presente estudo, obtiveram-se relativamente poucos casos em que foi realizado o acesso de Stoppa exclusivo. O uso da janela lateral do acesso ilioinguinal associado parece ser uma boa opção de acesso concomitante. Provavelmente, com o aumento da curva de aprendizado, será cada vez menos necessário o uso do acesso posterior de Kocher-Langenbeck no mesmo tempo, uma vez que estudos com amostras maiores apresentam menor porcentagem do uso concomitante desta via com o Stoppa modificado.

Acreditamos que a manutenção da integridade do canal inguinal propicia menos dissecção de partes moles e consequente menor chance de infecção. O tempo entre o trauma e a abordagem cirúrgica definitiva também pode ser considerado um fator importante, pois fraturas envelhecidas, com mais de 15 dias, tendem a ter uma menor mobilização e consequente difícil redução e, às vezes, com necessidade de mais de 2 acessos, com aumento do tempo cirúrgico e piores resultados funcionais.

## Conclusão

Conclui-se que, na coorte estudada, o acesso de Stoppa modificado proporcionou bons resultados clínicos funcionais, como demonstrado pela média de 92,5 no HHS modificado e 77,1% de excelentes ou bons resultados no MPS. O estudo da série de casos demonstrou relevância estatística positiva entre a qualidade da redução e os desfechos funcionais, e entre o tempo até a cirurgia e a qualidade da redução. No seguimento de um ano, demonstrou-se que o uso desta via pode ser uma excelente alternativa para as fraturas anteriores do acetábulo.
